# Influence of polarization angle on LIPSS formation and ablation efficiency in direct laser interference patterning of metals

**DOI:** 10.1038/s41598-025-07657-4

**Published:** 2025-06-25

**Authors:** Francisco Udo Marins Almeida, Bogdan Voisiat, Ignacio Tabares, Fabian Ränke, Andrés Fabián Lasagni

**Affiliations:** 1 Institute of Manufacturing, Technische Universität Dresden, George‑Bähr Str. 3c, 01069 Dresden, Germany; 2 Fraunhofer Institut für Werkstoff und Strahltechnik IWS, Winterbergstr. 28, 01277 Dresden, Germany

**Keywords:** Direct laser interference pattering, Laser induced periodic surface structures, Beam polarization, Ablation efficiency, Al2024, Stainless steel, Mechanical engineering, Ultrafast lasers

## Abstract

This study explores the influence of the polarization angle on the formation of Laser-Induced Periodic Surface Structures (LIPSS) during Direct Laser Interference Patterning (DLIP) and its impact on ablation efficiency in stainless steel and aluminum 2024 substrates. Two pulse durations, 12 ps and 70 ps, with a laser wavelength of 1064 nm, are employed at varying accumulated fluences to evaluate their effects on the surface structuring process. The results demonstrate that the Low Spatial Frequency LIPSS (LSFL) orientation with respect to the line-like structures produced by two-beam DLIP is strongly influenced by the polarization angle and the alignment of DLIP features. In addition, the spatial period of LSFL in stainless steel remained relatively stable regardless of the polarization angle (~ 900–1000 nm), whereas in aluminum 2024, it exhibited significant variation, decreasing from approximately 920 nm to 506 nm as the LSFL rotated. The polarization angle also affected the reached structure depth at constant irradiation conditions, particularly in stainless steel, where greater depths were achieved when the LSFL aligned perpendicularly to DLIP lines (over 50% variation). These findings provide valuable insights for optimizing laser-based surface processing techniques for metallic substrates.

**Supplementary Information:**

The online version contains supplementary material available at 10.1038/s41598-025-07657-4.

## Introduction

Laser beam polarization plays a crucial role in laser-matter interaction, significantly affecting optical absorption in highly reflective materials such as metals^1–4^. The absorption depends on both the polarization type (e.g., p or s) and the laser beam angle of incidence^5–7^influencing applications like laser cutting and material processing^1,8^.

In laser-based microstructuring, particularly with ultrashort pulsed lasers (fs and ps range), Laser-Induced Periodic Surface Structures (LIPSS) have been observed in various materials, including metals, semiconductors, and polymers^9–12^. In these cases, beam polarization determines the geometry and orientation of LIPSS, while laser wavelength primarily influences their spatial period.

A related structuring technique, Direct Laser Interference Patterning (DLIP), uses two or more overlapping laser beams to create periodic interference patterns on a material’s surface^13^. When the laser fluence exceeds the material removal threshold, ablation occurs at the interference maxima, forming a well-defined microstructure^14^. The resulting feature geometry (e.g., lines, dots, pillars) can be tailored by adjusting the number, orientation, and polarization of the interfering beams^15,16^. Since LIPSS formation is an intrinsic laser-material interaction phenomenon, these structures can also appear on DLIP-treated surfaces, particularly when using ultrashort pulses^17,18^. Moreover, controlling the beam polarization in DLIP has shown to influence the orientation of LIPSS^19^.

The combination of DLIP and LIPSS results in hierarchical surface structures with unique functional properties^20^. Schell et al. demonstrated this effect by producing line-like DLIP patterns (8.5 μm spatial period) on a Ti-13Nb-13Zr alloy, which also exhibited Low and High Spatial Frequency LIPSS (LSFL and HSFL) with spatial periods of ~ 600 nm and ~ 200 nm, respectively^21^. The LSFL aligned parallel to the DLIP lines, while the HSFL were perpendicular, consistent with laser polarization effects^22–26^. Similarly, Sikora et al. reported that when DLIP structures had spatial periods close to or smaller than the laser wavelength, electric field accumulation at DLIP peaks altered LIPSS topography, reducing its periodicity^27^. These interactions influence energy absorption, electric and magnetic field distributions, and, consequently, LIPSS morphology. Alamri et al. further explored the interplay between DLIP and LIPSS, demonstrating that when both structures have similar spatial periods, the dominant one depends on used the laser fluence. In particular, higher fluences favor DLIP, whereas lower fluences promote LIPSS features. Depending on the polarization direction, these structures may either compete or overlap^28^.

Such microstructured surfaces have broad technological applications. DLIP-processed surfaces with LIPSS can enhance wettability, friction, adhesion, and biocompatibility^29–34^. In tribology, these patterns optimize lubrication by reducing contact area and forming lubricant reservoirs, thereby reducing wear and extending lubricant lifespan^35–39^. In biomedical applications, nano- and microstructures influence cell adhesion, biocompatibility, and bacterial resistance, with sub-micrometer patterns shown to reduce bacterial adhesion through geometric constraints or ion release (e.g., Cu²⁺)^40–43^.

Despite extensive research on DLIP and LIPSS formation, the influence of beam polarization on ablation efficiency remains largely unexplored. Furthermore, it is unclear how polarization direction affects the orientation and spatial period of LIPSS formed at angles between 0° and 90°.

In this study, we investigate the role of polarization in DLIP microstructuring by producing line-like patterns on stainless steel and aluminum using a two-beam DLIP setup with ultra-short laser pulses (12 ps and 70 ps). These materials were chosen for their contrasting thermal properties, allowing us to assess how heat conduction influences polarization-driven structuring dynamics and ablation efficiency. The treated surfaces are analyzed using White Light Interferometry (WLI) and Scanning Electron Microscopy (SEM) to quantify both the morphology and the structure depth of the produced patterns. In this context, ablation efficiency is defined as the resulting depth of the line-like structures associated with the DLIP interference pattern, which are produced under constant process parameters.

## Materials and methods

For the laser structuring experiments, stainless steel (304) and aluminum (2024) plates were used. The initial surface roughnesses (Sa) were ~ 8 nm and 196 nm, respectively. Before laser structuring, all samples were cleaned with 99% (v/v) ethanol to remove contamination and subsequently dried with compressed air.

**Fig. 1:**
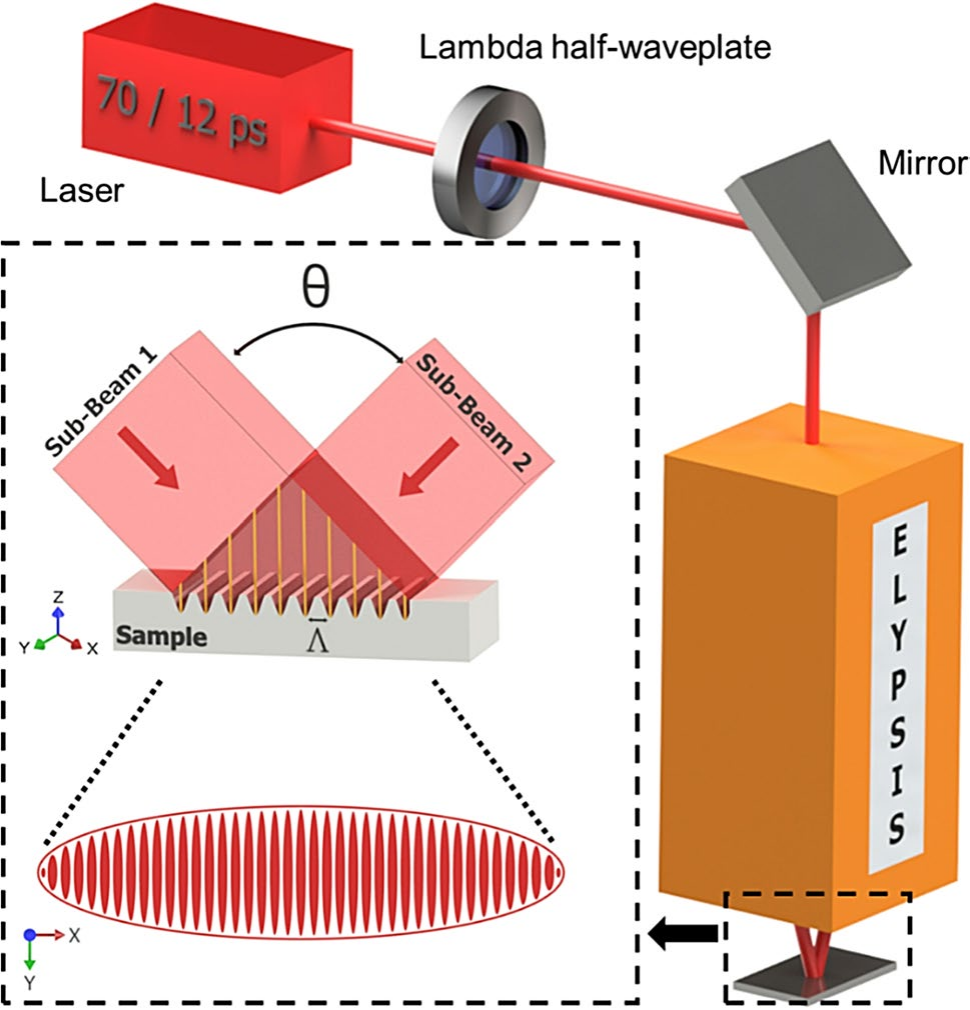
Experimental two-beam DLIP configuration (using the ELYPSIS^®^ optical head), showing the employed lambda half-wave plate for controlling beam polarization direction. The schematic of the elliptical beam spot that contains the line-like pattern geometry is shown in the inset (Image designed with Autodesk Inventor 2024, www.autodesk.com/products/inventor/overview).

The DLIP structures for both materials were fabricated using two different DLIP processing systems equipped with laser sources emitting pulses of 70 ps (neoMOS, neoLASE GmbH, Hannover, Germany) and 12 ps (PX200-3-GH, EdgeWave GmbH, Würselen, Germany) durations, both at 1064 nm wavelength. In both configurations, the laser beam is directed to an ELYPSIS^®^ (SurFunction GmbH, Germany) optical head, which splits the beam into two sub-beams that focus on a single spot, creating a line-line interference intensity profile with periods (Λ) of 5.4 μm and 6.0 μm for the 70 ps and 12 ps setups, respectively. In both setups, a zero order half-wave plate was placed before the optical head in order to change the laser beam polarization during the experiments. The used configuration for both laser sources is shown in Fig. 1.

For the laser texturing experiments, the laser fluence was varied between 1.1 and 1.7 J/cm^2^. The pulse-to-pulse overlap (in the direction parallel to the interference lines, corresponding to the sample movement) was also adjusted, resulting in accumulated fluences from approximately 11 to 260 J/cm^2^. The process parameters used in this research work are listed in Table 1, including the size of the resulting elliptical spots for both used configurations. For each used accumulated fluence, the polarization angle was varied from 0° to 90° in 10° intervals. The starting angular position of the lambda half-wave plate was selected corresponding to a polarization angle parallel to the interference lines of the two-beam DLIP configuration. This position is referred to as 0° in this study, with the polarization angle increasing up to 90°, where it becomes perpendicular to the DLIP interference lines. To differentiate the polarization angle from the LIPSS orientation, the LIPSS angle is defined as -90°, corresponding to structures perpendicular to the DLIP interference lines, and increases up to 0°, where the LIPSS are parallel to the DLIP lines. The scanning strategy used in this study was selected over stationary (fixed-point) irradiation, as it more accurately reflects the practical requirements of large-area surface functionalization. This approach enables the treatment of surfaces significantly larger than the laser spot size and is therefore more relevant for industrial-scale applications.

**Table 1:** Process parameters used in this study, including laser fluence and accumulated fluence as well as the size of the elliptical DLIP spots for 12 and 70 Ps pulse durations.

Pulse duration (τ) (ps)	Spot size (µm x µm)	Laser fluence (Φ) (J/cm^2^)	Accumulated fluence (Φ_acc_) (J/cm^2^)
12	61 × 793	1.1	11.2
			22.4
			56.1
			112.0
70	136 × 359	1.7	82.2
			160.0
			225.0
			262.0

Surface characterization was performed using a White Light Interferometer (WLI) (Sensofar S Neox, Terrassa, Spain) equipped with a 50x magnification objective, providing lateral and vertical resolutions of 500 nm and 1 nm, respectively. Each fabricated structure was measured three times in order to provide statistical significance. Morphological analysis was conducted using two Scanning Electron Microscopes (SEM) from Carl Zeiss, Germany: Supra 40VP and Sigma 300 Gemini operated at acceleration voltage of 5 kV, and 3 kV, respectively. Fast Fourier Transformations (FFT) were applied to each SEM image to analyze the spatial frequency and orientation of the LIPSS. In the case of LIPSS, the FFT typically reveals two diffuse intensity clouds, due to the quasi-periodic nature of these features. The angular position of these clouds with respect to the vertical axis can be used to extract the orientation angle of the LIPSS. In the case of Al2024, the LIPSS were not very pronounced, and the FFT did not produce clearly discernible features that could be reliably analyzed. For each condition, three independent measurements were taken to ensure consistency. For periodicity, two points were selected with at least five features (either HSFL or LSFL) between them, and the measured distance was divided by the number of features along the line.

## Results and discussion

To specifically isolate the influence of polarization angle on the DLIP structuring process, all irradiation parameters, including accumulated fluence, number of pulses, and beam overlap, were kept constant for each condition, with the exception of the polarization angle. Consequently, the initial analysis focuses on the resulting structure depth as a direct indicator of ablation behavior under fixed irradiation conditions.

The polarization angle was systematically varied for both pulse durations by rotating the half-wave (λ/2) plate in 5° increments, corresponding to a 10° change in the beam polarization direction. This is because the output polarization is twice the angle between the input polarization and the half-wave plate axis. In each experiment, 5 mm long single-lines areas were treated, allowing for the measurement of average structure depths resulting from material ablation at the interference maxima due to the DLIP process^44^.

**Fig. 2:**
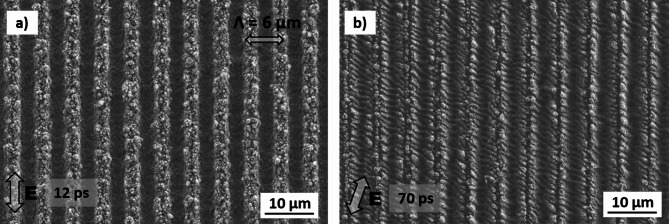
Exemplary SEM images of DLIP treated (a) aluminum 2024 and (b) stainless steel substrates. The used process conditions were: (a) *Φ*_*acc*_ = 22.4 J/cm^2^, 12 ps, (b) *Φ*_acc_ = 160.0 J/cm^2^, 70 ps.

Figure 2a and b show the resulting DLIP features on aluminum 2024 and stainless steel 304, respectively, denoting a high degree of uniformity for the used process parameters. The polarization direction (vector of the electric field *E*) is indicated by a double arrow in each image. These SEM images confirm that the DLIP-induced line-like surface structures are homogeneously distributed over large areas for both materials. In the following sections, a more detailed analysis of the pattern morphology and structure orientation as a function of polarization angle is presented, starting with stainless steel, followed by Al2024.

**Fig. 3:**
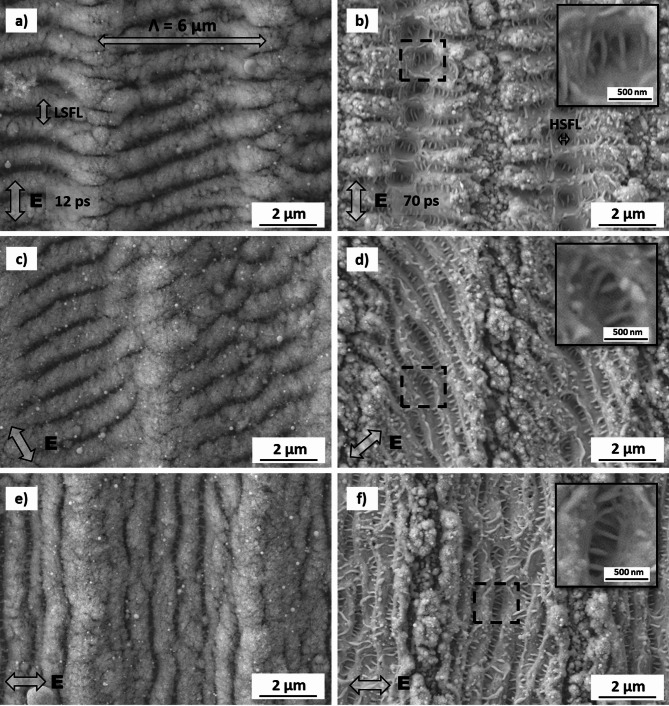
High-resolution SEM images of laser-treated stainless steel samples with different polarization angles for 12 ps (left) and 70 ps (right) pulse durations. The used accumulated fluences were: (a, c, e) *Φ*_*acc*_ = 22.4 J/cm^2^; (b, d, f) *Φ*_*acc*_ = 160.0 J/cm^2^.

Figure 3 shows representative high-resolution SEM images of stainless steel samples treated at different pulse durations, polarization angles, and accumulation laser fluences. Also, in this case, the polarization direction is indicated in the figures. The images on the left and right side were produced at 12 ps and 70 ps pulse durations, respectively (what is also indicated in each image). The spatial period of the DLIP features (6.0 μm) in the 12 ps pulse configuration (Fig. 2a), is in agreement with the used optical configuration.

Under most combinations of pulse duration and laser fluence, the following features were consistently observed. First, the DLIP features are covered by (quasi) periodic ripple-like surface structures that can be identified as LIPSS. As shown in Fig. 3, for the 12 ps pulses (Fig. 3a, c and e), LIPSS features are covering the entire DLIP-structured area, whereas for the 70 ps treatment, the formation of LIPSS is restricted to the interference maxima positions (Fig. 3b, d and f). Additionally, particle redeposition is observed at the interference minima positions for both pulse durations. For the applied pulse duration of 70 ps, High Spatial Frequency LIPSS (HSFL) were also observed (indicated as HSFL in Fig. 3b, see also insets in 3b, 3d and 3f), oriented perpendicular to the LSFL. Their average spatial period was approximately 200 nm. Further high-magnification SEM images of these structures are shown in the supporting information section (Fig. S3).

For both pulse durations, LSFL generally align perpendicularly to the polarization direction, which is typical for several metals^24,34^. However, this is not consistent for all polarization angles. For example, in Fig. 3a and b, where the polarization direction (*E*) was 0° (parallel to the DLIP pattern), LSFL formed almost perpendicular to the DLIP features. In Fig. 3c and d, with the polarization angles of 30° and 50°, the LIPSS orientation was significantly affected, but they were not perpendicular to *E*. In contrast, Fig. 3e and f show that at *E* = 90°, LSFL were perpendicular to the polarization direction and parallel to the DLIP features.

**Fig. 4:**
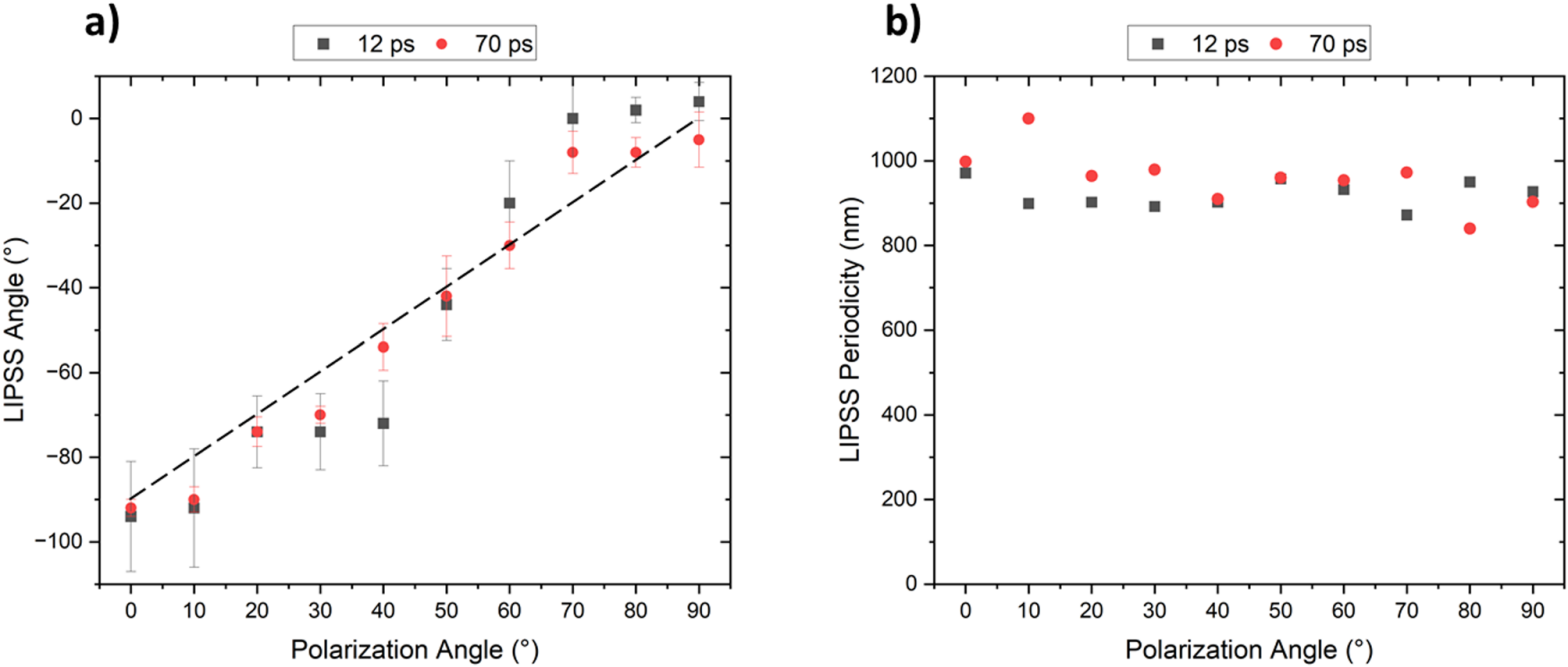
(a) LSFL orientation angle (12 ps and 70 ps pulse durations) according to the polarization vector angle. An angle of – 90° means that the LSFL are perpendicular to the DLIP lines, while 0° represents the situation when the LSFL are parallel to the DLIP periodic features. The dashed line represents the expected LIPSS orientation under the assumption that the structures form exactly perpendicular to the polarization vector. (b) Spatial period Λ_LSFL_ of the LSFL dependence on the polarization angle. Material: stainless steel. The accumulated laser fluence was *Φ*_*acc*_ = 22.4 J/cm^2^ for 12 ps and *Φ*_*acc*_ = 160.0 J/cm^2^ for 70 ps.

To further investigate this effect, Fig. 4a plots the average LSFL orientation as a function of the polarization angle. The dashed line represents the expected LIPSS orientation under the assumption that the structures form exactly perpendicular to the polarization vector. As observed, for polarization angles between 0° and 40°, the LSFL orientation changed only from − 94° to -72° (for the 12 ps pulses), which represent only a total rotation of 22°, that is 18° less than the polarization rotation. The reported angles were calculated from FFT analysis of the SEM images, as described in the Experimental section. The corresponding transformations are also provided in the Supporting Information (Fig. S1). For a polarization angle of 50°, the average angle of the LSFL represents − 44°, corresponding to a total rotation of 42° (8° less than expected). This indicates that the DLIP lines restrict first LIPSS rotation, preventing them from being perpendicular to the polarization vector at all angles. For larger polarization angles, the opposite behavior was observed. For example, at a 70° angle for the polarization vector, the LSFL orientation angle was 0°, which means that the LSFL rotated in total 94° (20° more than the polarization vector). Thus, beyond a certain angle (~ 50°), this relationship shifts abruptly, positioning the LIPSS above the expected orientation (orientation angle above the dash line shown in Fig. 4a). In other words, LSFL tend to align more closely with either the parallel or perpendicular direction of the DLIP lines. Finally, at 90° of the polarization angle, the LSFL showed almost a parallel orientation compared to the DLIP lines (approx. ~4° difference). This behavior was also observed for the pulse duration of 70 ps, as shown in Fig. 4a (see also Supporting Information, Fig. S2).

The observed reorientation behavior of LSFL with changing polarization angles can be explained by the interplay between the laser’s electric field direction and surface plasmon polariton (SPP) propagation. SPPs are preferentially excited along directions parallel to the incident electric field, and their interference with the incoming beam defines the spatial energy modulation that drives ripple formation. As the polarization vector rotates, the SPP propagation direction rotates accordingly, and consequently the LSFL orientation^9,39^. However, this idealized relationship can break down due to additional surface-related factors. In particular, recent studies have demonstrated that pre-existing surface features, such as polishing-induced grooves or anisotropic roughness, can influence LIPSS orientation by introducing directional scattering and modifying local field enhancement^46,47^. When the polarization vector forms an intermediate angle with respect to these surface anisotropies (e.g., below ~ 50°), the LSFL may form at a compromise angle, reflecting a balance between polarization-guided plasmonic effects and surface-guided scattering. Furthermore, Sotelo et al. showed that for rougher metallic surfaces, ripple regularity and orientation are increasingly governed by the dominant topographic direction, especially when the roughness exceeds threshold values (e.g., Sa > 200 nm)^48^. This competition can lead to gradual misalignment or abrupt reorientation of LSFL, as observed in this study.

It is important to note that, unlike in studies where LIPSS formation is influenced by pre-existing surface anisotropy (e.g., polishing marks or mechanical grooves), the substrates in this work were initially flat and featureless prior to irradiation. In our case, the surface roughness that may affect LIPSS orientation is generated in situ by the early stages of the DLIP process itself. This suggests that the periodic modulation imposed by the interference pattern occurs early and rapidly enough to influence the electromagnetic feedback mechanisms responsible for LSFL formation.

The average LSFL spatial periods were measured from SEM images as a function of polarization angle. As shown in Fig. 4b, the LSFL period remained largely unaffected by polarization angle for samples irradiated with 12 ps pulses at an accumulated fluence of 22.4 J/cm^2^ and 70 ps pulses at 160.0 J/cm^2^. The period ranged from 820 nm to 1100 nm for both pulse durations, slightly smaller than the laser wavelength (1064 nm), which is typical for LSFL on various metals^27,49,50^. The average spatial periods of both LSFL and HSFL for stainless steel 304 and both pulse durations are summarized in Table 2.

**Table 2:** Measured spatial period range of both LSFL and HSFL for steel and aluminum 2024, depending on the pulse duration from polarization vector angles 90° (minimum) to 0° (maximum).

Material	Pulse duration (ps)	LIPSS	Spatial period 0°-90° (nm)
Stainless steel 304	12	LSFL	820–1015 nm
		HSFL (*)	241–300 nm
	70	LSFL	840–1100 nm
		HSFL	198–203 nm
Aluminum 2024	12	LSFL	not visible
		HSFL	not visible
	70	LSFL	506–920 nm
		HSFL	not visible

**Fig. 5:**
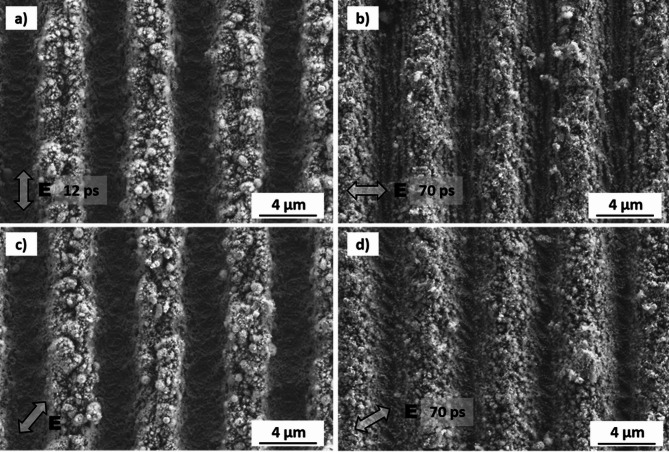
High resolution SEM images of laser treated aluminum 2024 samples, showing the line-like DLIP features with a spatial period of 6.0 μm (left) and 5.4 μm (right) for different polarization angles and pulse duration of 12 ps (left) and 70 ps (right). The used accumulated fluences were: (a, c) *Φ*_*acc*_ = 22.4 J/cm^2^; (b, d) *Φ*_*acc*_ = 160.0 J/cm^2^.

A different behavior was observed for aluminum 2024. As shown in Fig. 5a and c, for the 12 ps pulses, no LIPSS features (neither LSFL nor HSFL) were observed at an accumulated laser fluence of 160 J/cm^2^, regardless of the polarization vector orientation (indicated also in this case as E in the SEM images). In comparison, for 70 ps pulses (Fig. 5b and d), LSFL features were visible, but not HSFL. Recent findings suggested that the formation of LSFL/HSFL is uncommon for this material. LSFL have been primarily observed when using fs laser pulses when structuring in air, while HSFL have been produced, for example, when structuring aluminum in a liquid environment^24^. Other studies have shown that ablated regions on Al exhibit a random distribution of features (also when using ~ 70–300 fs pulses)^42,43^and also that for lower fluences, LIPSS form at the center of the laser spot (having a Gaussian intensity distribution) with random orientation, and that they expand to borders of the spot with increasing number of applied pulses^51,52^.

**Fig. 6:**
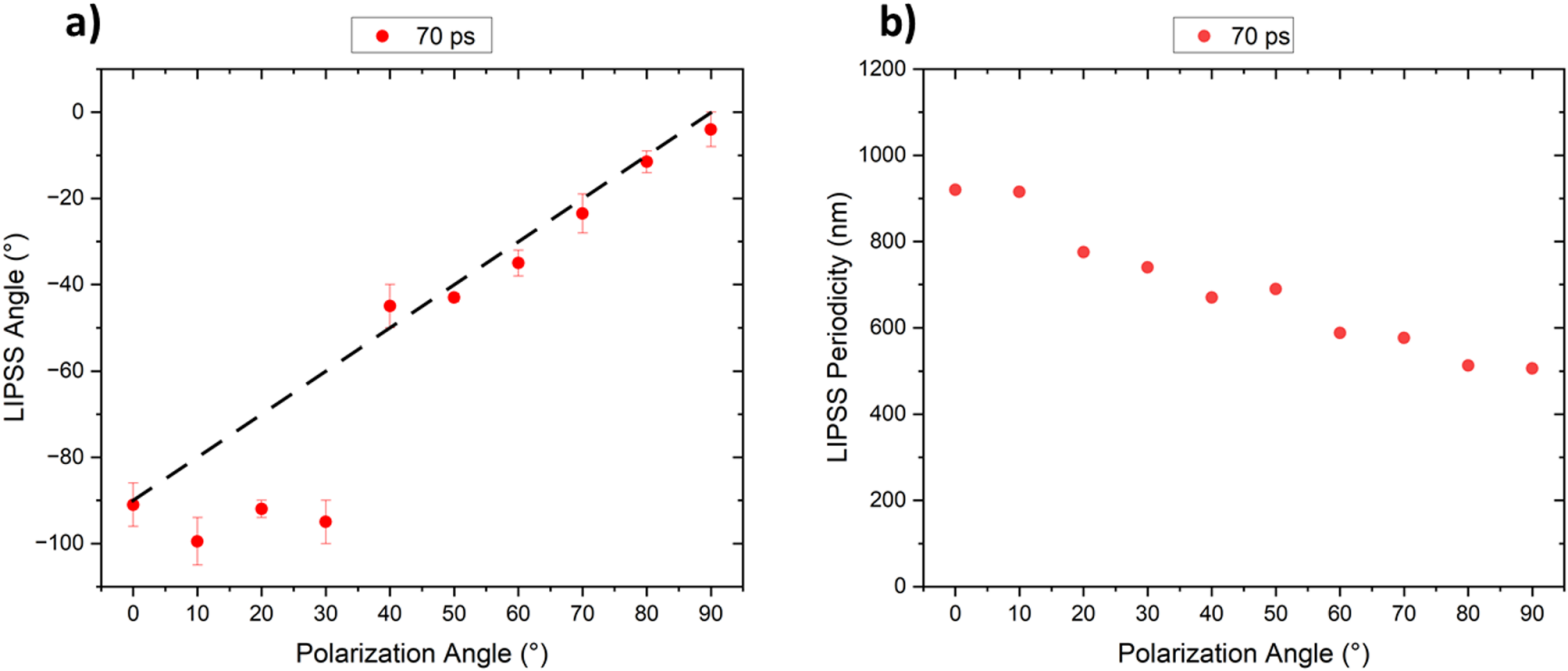
(a) LSFL orientation angle (70 ps) relative to the polarization vector angle. (b) Spatial period of the LSFL as a function of the polarization vector angle. The dashed line represents the expected LIPSS orientation under the assumption that the structures form exactly perpendicular to the polarization vector. Material: aluminum 2024. The accumulated laser fluence was *Φ*_*acc*_ = 160.0 J/cm^2^.

Similarly to the steel surfaces, for the aluminum 2024 samples irradiated with 70 ps pulses, the dependency of LSFL orientation on the polarization vector was also evaluated. The results are shown in Fig. 6a. For polarization vector angles between 0 and 40°, the produced LSFL were almost perpendicular to the DLIP lines, with angles ranging from − 90° to -100° (see Figs. S3 and S4 in Supporting Information section), in indicating that the DLIP lines exert a stronger influence in this case. When the polarization angle reached 40°, a rapid rotation of the LSFL from − 90° to approximately − 45° was observed. Further increasing the polarization angle led to a greater rotation of the LSFL, reaching − 4° at a polarization angle of 90°. This corresponds to a total rotation of 86°, with the LIPSS orientation becoming substantially perpendicular to the polarization vector. Thus, it can then be concluded that for angles below 40° of the polarization vector, the LIPSS present a strong resistance to rotate, maintaining their orthogonality to the DLIP. For angles greater than 40°, the LIPSS rotation angle becomes nearly perpendicular to the polarization vector (see also the dashed line in Fig. 6a, which represents the expected LIPSS orientation assuming the structures form exactly perpendicular to the polarization vector), with the DLIP lines having relatively little influence. As mentioned before, the interplay between the DLIP interference pattern and LIPSS orientation, driven by surface plasmon polariton excitation, plays a key role in determining the final surface morphology. In the case of Al2024, this coupling appears to be less stable or more sensitive to polarization angle, potentially due to the material’s higher thermal conductivity and weaker electron–phonon coupling, which can disrupt the regular formation of ripples^25^.

Regarding the spatial period of the LSFL, changing the orientation of the polarization vector caused a significant variation in the period, as shown in Fig. 6b. For example, at 0° (where the LSFL are perpendicular to the DLIP lines), the spatial period was approximately 920 nm, which corresponds to about 90% of the laser wavelength used (1064 nm) and is typical for LSFL, as previously observed for steel. However, as the polarization vector angle increased from 0° to 90°, the spatial period steadily decreased to about 506 nm (a ~ 45% reduction), despite the LSFL angle remaining almost unchanged from 0° to 40°. This behavior can be attributed to changes in the mean free path of surface plasmon polaritons, as their interaction with the periodic DLIP structures alters the local electromagnetic field and modifies the conditions for plasmon excitation and propagation^27,49^. For instance, a similar reduction in LSFL spatial period has been experimentally observed in stainless steel under DLIP conditions (with a spatial period of 1.48 μm) when the polarization direction was perpendicular to the interference fringes. In this mutual interaction regime, the LSFL period dropped significantly from conventional values (~ 800–900 nm) to approximately 470 nm (or ~ 0.44λ), well below the theoretical predictions for standalone LIPSS formation^27^. This effect was attributed to strong coupling between the DLIP-induced periodic field and surface plasmon polariton (SPP) excitation, resulting in highly regular ripple formation with low angular dispersion. However, why this effect did not manifest in the steel samples under identical conditions remains unclear. A possible explanation could be the significantly larger DLIP spatial period used in our experiments (6.0 μm compared to ~ 1.48 μm in the study by Sikora et al.), which may weaken the field coupling between the interference pattern and surface plasmon excitation. This hypothesis requires further investigation, potentially through numerical simulations of the electromagnetic and thermodynamic interactions during laser irradiation. The average spatial periods of LSFL for aluminum 2024 and both pulse durations are also summarized in Table 2.

**Fig. 7:**
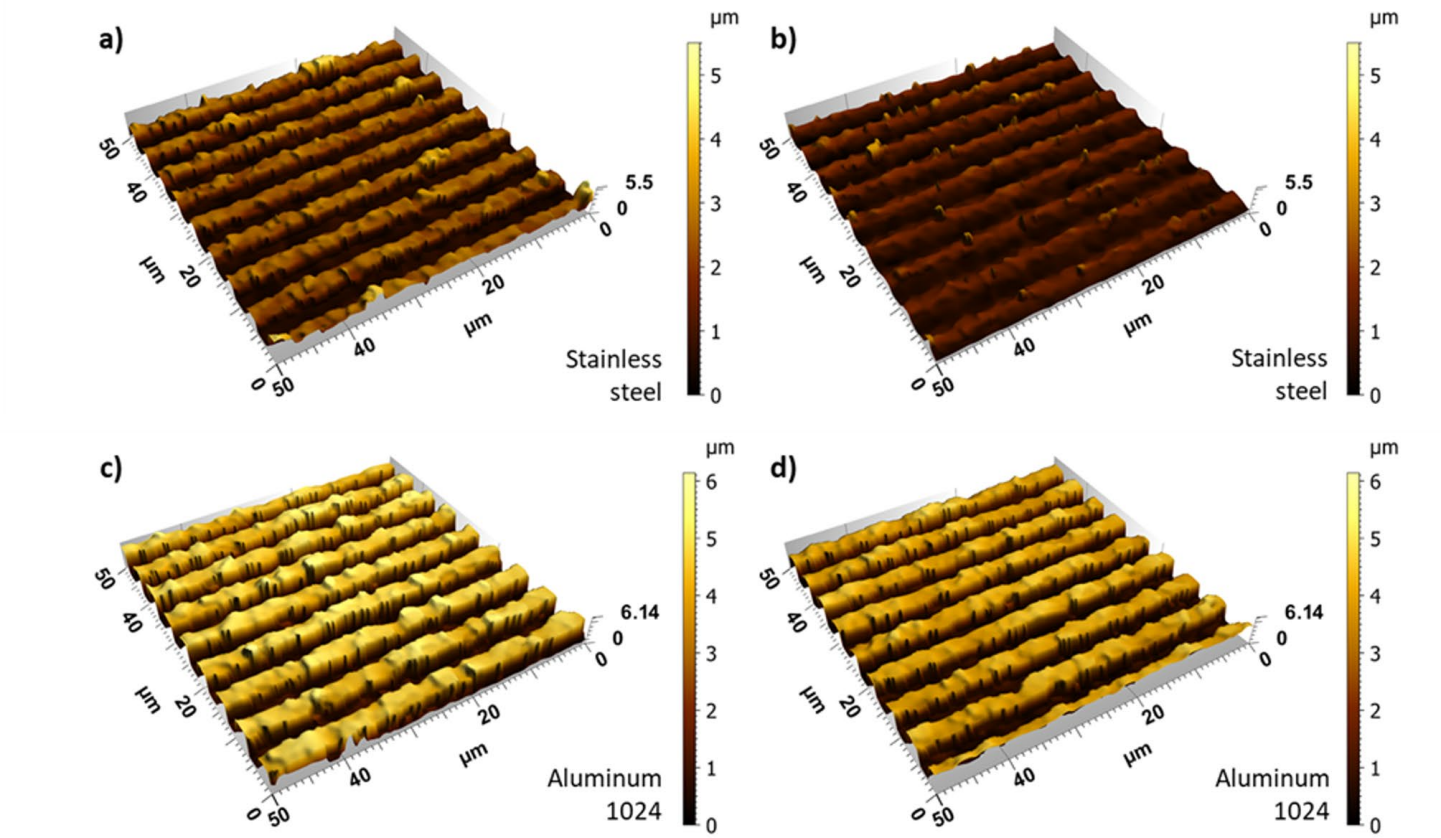
Exemplary WLI images of patterned stainless steel (a, b) and aluminum 2024 (c, d) samples using 12 ps pulses at accumulation fluences and polarization angles of: (a) *Φ*_*acc*_ = 56.1 J/cm^2^, 0°; (b) *Φ*_*acc*_ = 56.1 J/cm^2^, 90°; (c) *Φ*_*acc*_ = 22.4 J/cm^2^, 0°; (d) *Φ*_*acc*_ = 22.4 J/cm^2^, 90°. (Topography acquired via WLI and analyzed in SensoMAP Premium Version 7.4.9, www.sensofar.com/metrology/software/sensomap/)

Following the preceding analysis of LIPSS orientation and spatial period, the influence of polarization angle on the depth of the DLIP line-like textures was further investigated. Structure depth was quantified using White Light Interferometry (WLI). Exemplary measurements for stainless steel treated with 12 ps pulses and an accumulation fluence of 56.1 J/cm² at 0° and 90° polarization angles are shown in Fig. 7a and b, respectively. Similarly, DLIP structures produced on aluminum 2024 are shown in Fig. 7c and d, also treated with 12 ps pulses and an accumulation fluence of 22.4 J/cm², at 0° and 90° polarization angles, respectively.

The results indicate that the polarization angle, and consequently the orientation of the LIPSS, significantly effects the resulting structure depth and, thus, the efficiency of the texturing process. For example, in the surface topographies shown in Fig. 7a and b for stainless steel, the measured structure depths were 3.7 μm and 2.4 μm at polarization angles of 0° and 90°, respectively, indicating 54% deeper DLIP structures in the first case (when keeping all other process parameters constant). In other words, when the LSFL were oriented perpendicularly to the DLIP lines, the structure depth increased significantly. In contrast, for the conditions shown in Fig. 7c and d for aluminum 2024, the structure depths were 4.5 μm and 3.9 μm with a difference of only 15%, for 0° and 90° angles. The following paragraphs provide a detailed discussion of the results for both laser-treated materials.

**Fig. 8:**
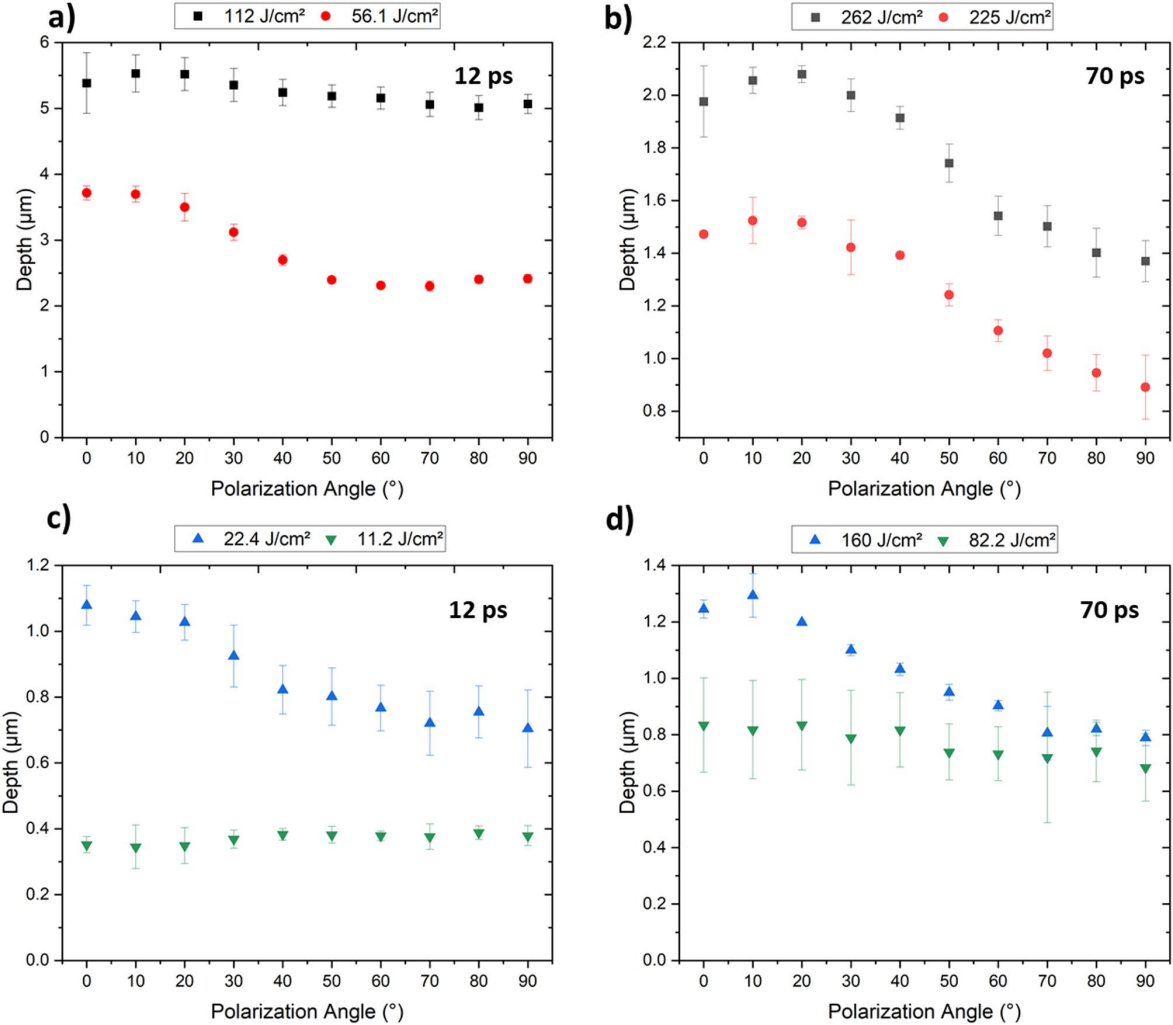
Dependency of the achieved structure depth of the DLIP features on the polarization angle for stainless steel. The process parameters used are indicated in the images: (a, c) 12 ps and (b, d) 70 ps pulse durations (for a 0° polarization angle, the LSFL are perpendicular to the DLIP line features).

Figure 8 shows the evolution of the structure depth of the DLIP features in stainless steel as a function of polarization angle and accumulated laser fluence for pulse durations of 12 ps and 70 ps. The following observations are possible when comparing the obtained results depending on the accumulated laser fluences and pulse durations. The 12 ps laser pulses produced, in general, deeper structures, even when using lower accumulated laser fluences, above the threshold of *Φ*_*acc*_ = 22.4 J/cm^2^ (see Fig. 8a, c). At relatively low accumulated laser fluences, the reached structure depths did not show any significant variation with the polarization angle. For example, at 11.2 J/cm² for the 12 ps pulses (Fig. 8c), the structure depth was approximately 0.35 μm for all polarization angles. For the 70 ps, the structure depth was 0.75 μm at *Φ*_*acc*_ = 82.2 J/cm^2^ (Fig. 8d) and also did not significantly change with the angle of polarization.

However, for accumulated laser fluences between 160.0 J/cm² and 260.0 J/cm^2^ with 70 ps pulses, and between 22.0 J/cm² and 56.0 J/cm^2^ for the 12 ps pulses, the structure depth was significantly larger at 0°, corresponding to the angle in which the LSFL features are perpendicular to the DLIP lines. For example, at 12 ps and 22.4 J/cm², the structure depths were ~ 1.1 and ~ 0.8 μm at 0° and 90° polarization angles, respectively, representing a difference of ~ 38% (Fig. 8c). At 225.0 J/cm² and 70 ps pulse duration (Fig. 8b), the structure depths were ~ 1.5 μm and ~ 0.9 μm for the polarization angles mentioned above, also showing a significant difference (structure depth difference of 67%).

To explore whether polarization-dependent absorption at the incidence angle imposed by the DLIP setup (considering a 5.1° incident angle required to obtain a 6.0 μm spatial period) could explain the observed differences in ablation efficiency, the reflectance for both s- and p-polarizations at the above mentioned angle was calculated for stainless steel and aluminum. The results showed that the difference in reflectance between polarization states was minimal (less than 0.2% for stainless steel and nearly negligible for aluminum). This indicates that angular absorption anisotropy is not a significant contributing factor at these shallow incidence angles. Therefore, the observed polarization-dependent differences in structure depth and ablation efficiency are more likely attributable to mechanisms such as localized field enhancement from interference-LIPSS coupling, differences in material response to surface plasmon excitation, or polarization-induced modulation of heat diffusion and melt dynamics.

When the highest accumulation fluence was applied for the 12 ps (112.0 J/cm^2^), the deepest structures were produced (~ 5.5 μm, with 6.0 μm of spatial period). However, the effect on the polarization angle on the structure depth was negligible (Fig. 8a). As shown by Ränke et al., ablation during DLIP processing of materials with high accumulated fluences leads to the ejection of material particles, which subsequently re-deposit from the ablation regions (intensity maxima) onto the peaks (intensity minima) positions of the DLIP pattern. This re-deposition significantly influences the structural formation of the DLIP pattern, playing a dominant role that overshadows the effect of the polarization angle^53^.

**Fig. 9:**
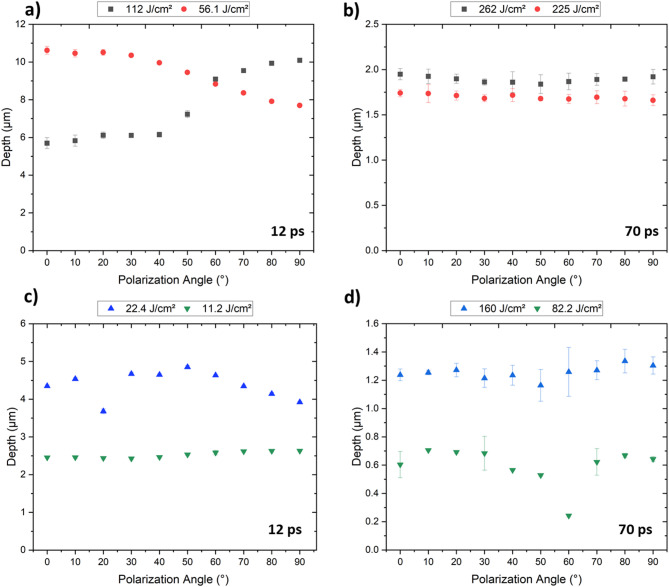
Dependency of the achieved depth to the polarization angle on aluminum 2024 for the DLIP configurations using 12 ps (a and c) and 72 ps (b and d) pulses.

In the case of the aluminum 2024 treated samples, the variation in the polarization angle did not significantly affect the structure depth of the fabricated DLIP structures for most used conditions (see Fig. 9). Only the samples treated with 12 ps pulses and accumulated fluences of 56.1 and 112.0 J/cm^2^ presented relevant differences (Fig. 9a). For the sample treated at 56.1 J/cm^2^, a behavior similar to that observed in stainless steel was seen, with structure depths of approximately 7.7 μm and 10.6 μm (a 27% difference) for the 90° and 0° polarization, respectively. In contrast, for 112.0 J/cm^2^, the opposite behavior was observed. As mentioned earlier, higher accumulated laser fluences, which result in structures with high aspect ratios, lead to significant re-deposition of material^53^.

**Fig. 10:**
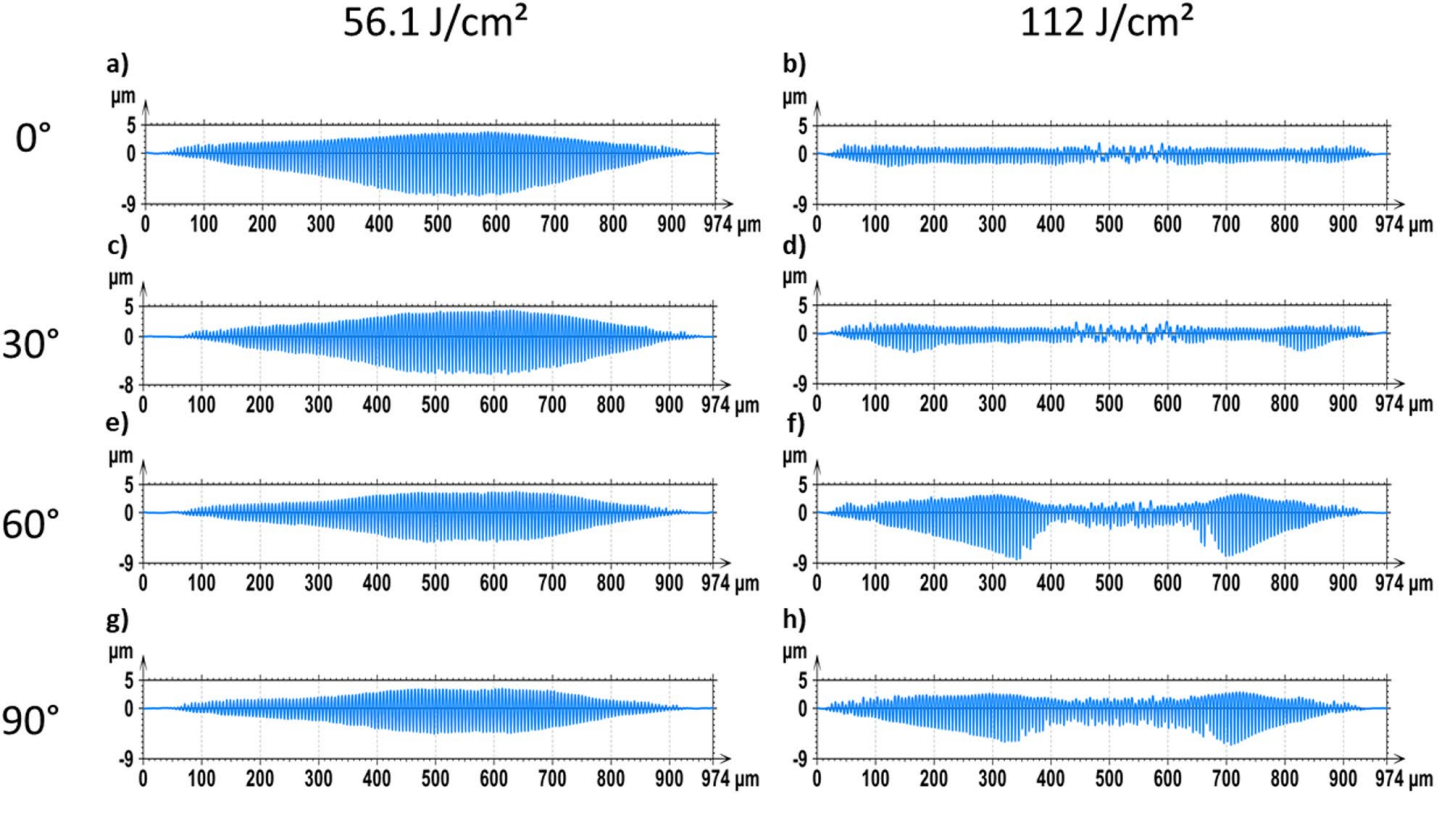
Surface profiles of the DLIP treated Al2024 samples surfaces with 12 ps duration, at accumulated fluences of *Φ*_*acc*_ = 56 J/cm^2^ and *Φ*_*acc*_ = 112 J/cm^2^ (right), for polarization angles of 0° (a, b), 30° (c, d), 60° (e, f) and 90° (g, h).

To confirm the last hypothesis, surface profiles of irradiated Al2024 samples, depending on polarization angle and cumulative laser fluence, are plotted in Fig. 10. For the samples irradiated with an accumulated fluence of 56 J/cm² (Fig. 10a), which seems to be below the cumulative fluence threshold for collapse, the structure depth decreases as the polarization vector orientation angles changes from 0° to 90°. In addition, for almost all conditions, the deepest features are obtained at the central area of the DLIP spot, due to the Gaussian intensity distribution of the laser beam profile. In the case of the Al2024 surface irradiated at 112 J/cm^2^ (Fig. 10b), this behavior is only observed at the more external regions of the laser spot, while at the central region, the depth of the features at the interference maxima positions is significantly smaller, as the ablated material redeposits at these positions^53^. This means that although the polarization vector orientation of 0° is generally beneficial for generating deeper structures, it does not always lead to a higher ablation efficiency, as the ablated material may not be fully removed from the surface, but rather redeposits at the center of the ablation area, when working at high cumulated fluences.

## Conclusions

In this study, line-like DLIP structures were produced on stainless steel and aluminum 2024 substrates by changing the polarization angle. Two different laser sources were used, delivering 12 ps and 70 ps pulses, both with a wavelength of 1064 nm, with various values of accumulated fluence. The following findings resulted from the performed experiments:

I. For stainless steel, both LSFL and HSFL were observed. The last specially for 70 ps pulses.
II. The LSFL orientation was slightly out of place regarding the polarization vector orientation, especially for polarization angles below 50°. For larger angles, the LSFL orientation angles abruptly increases tending to be perpendicular to the DLIP lines. This deviation suggests a competition between the polarization-driven orientation of LIPSS and the spatial modulation imposed by the DLIP interference pattern, with the latter exerting a stronger influence at lower angles.
III. In aluminum 2024, LSFL were only observed for the 70 ps pulses, while no LIPSS structures were present when using a pulse duration of 12 ps.
IV. The LSFL orientation in aluminum was more strongly influenced by the DLIP lines compared to stainless steel, being almost parallel to the DLIP lines until a 40° polarization angle. For larger angles, an abrupt change in the rotation behavior was observed for the LSFL, and they were almost perpendicular to the polarization angle. This indicates a stronger coupling between the DLIP-induced interference field and LIPSS formation in Al2024, potentially due to material-specific properties such as higher thermal conductivity.
V. For stainless steel, the LSFL spatial periods ranged between 810 nm and 1020 nm, closely matching the laser wavelength (1064 nm), and were not significantly influenced by the polarization angle.
VI. In aluminum 2024, LSFL spatial periods exhibited strong variation with rotating polarization, in the range from 506 nm to 920 nm as the LSFL rotated, suggesting a stronger interaction between DLIP patterning and LIPSS formation. This pronounced variation suggests that mutual interaction between the DLIP field and surface plasmon excitation modulates the effective ripple period, especially under polarization conditions that reinforce field confinement.
VII. Finally, the polarization angle has shown to have a significant effect on the structure depth of the DLIP features, especially for stainless steel. In general, structure depths were significantly greater when the LSFL were oriented perpendicularly to the DLIP lines.

These results underline the importance of understanding material-dependent effects in polarization-controlled DLIP processing, as they help define the limits and opportunities for tailoring surface morphology. In particular, despite the reduced LIPSS formation in Al2024, the observed differences in structure depth highlight that polarization remains a relevant parameter for optimizing ablation efficiency.

## Electronic supplementary material

Below is the link to the electronic supplementary material.

Supplementary Material 1

## Data Availability

Data will be made available on request to the Corresponding Author.
