# Supplementary material for: Influence of polarization angle on LIPSS formation and ablation efficiency in direct laser interference patterning of metals
Source: Sci Rep. 2025 Jun 25;15:20285. doi: 10.1038/s41598-025-07657-4 (PMC12198375; doi:10.1038/s41598-025-07657-4)
Supplement: Supplementary file 1 — Supplementary Material 1 [file 41598_2025_7657_MOESM1_ESM.pdf]

Influence of Polarization Angle on LIPSS Formation and Ablation Efficiency in Direct Laser Interference Patterning of Metals

Francisco Udo Marins Almeida[1], Voisiat, Bogdan[1], Ignacio Tabares[1], Fabian Ränke[1], Andrés Fabián Lasagni[1,2*]

[1] Institute of manufacturing, Technische Universität Dresden, George-Bähr Str. 3c, 01069 Dresden, Germany

[2] Fraunhofer Institut für Werkstoff und Strahltechnik IWS, Winterbergstr. 28, 01277 Dresden, Germany

* E-mail: andres_fabian.lasagni@tu-dresden.de

Supporting information

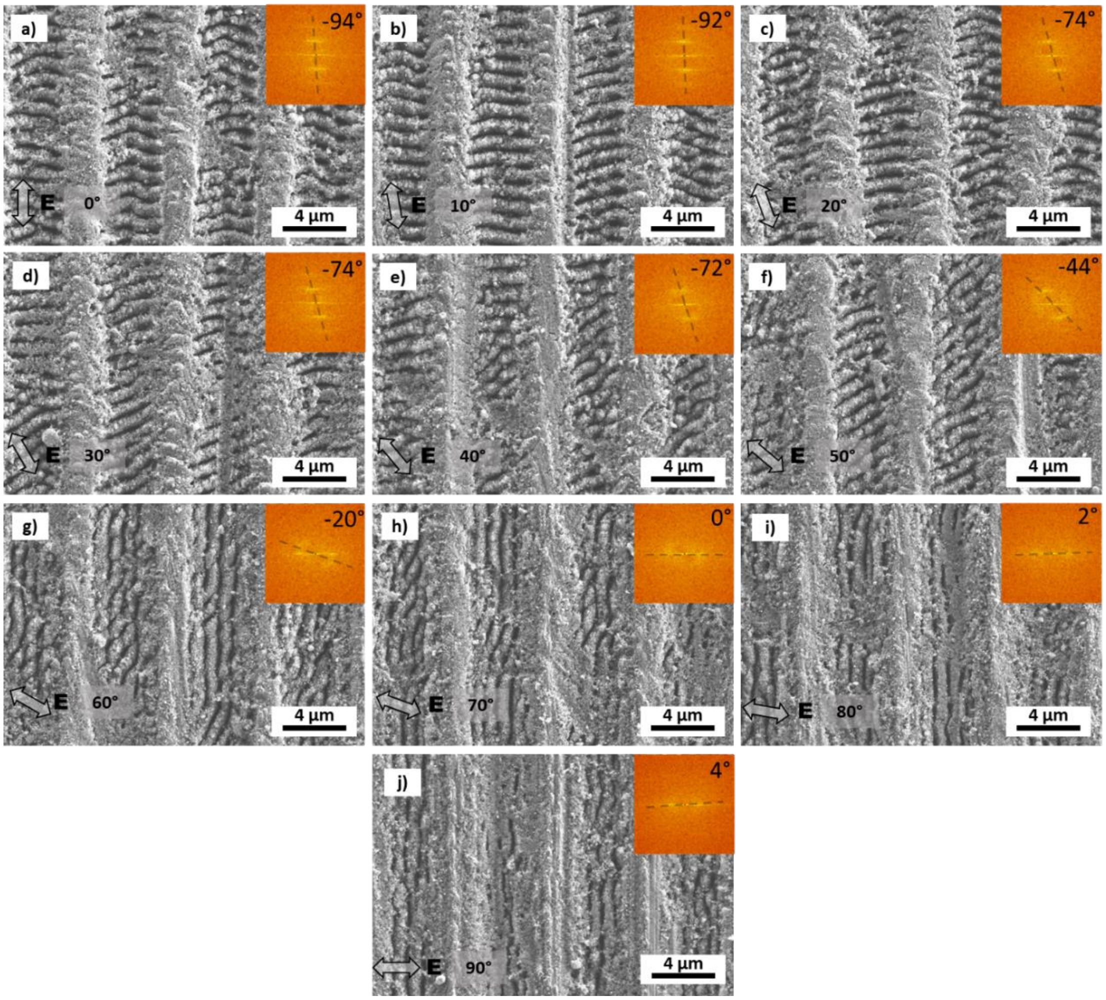

Figure S1: DLIP Stainless Steel surfaces treated with 12 ps pulses and 22.4 J/cm² fluence at different polarisation orientation angles; (a) 0°; (b) 10°; (c) 20°; (d) 30°; (e) 40°; (f) 50°; (g) 60°; (h) 70°; (i) 80°; j (90°), with respective insets showing the FFT corresponding to the image and the measured angle.

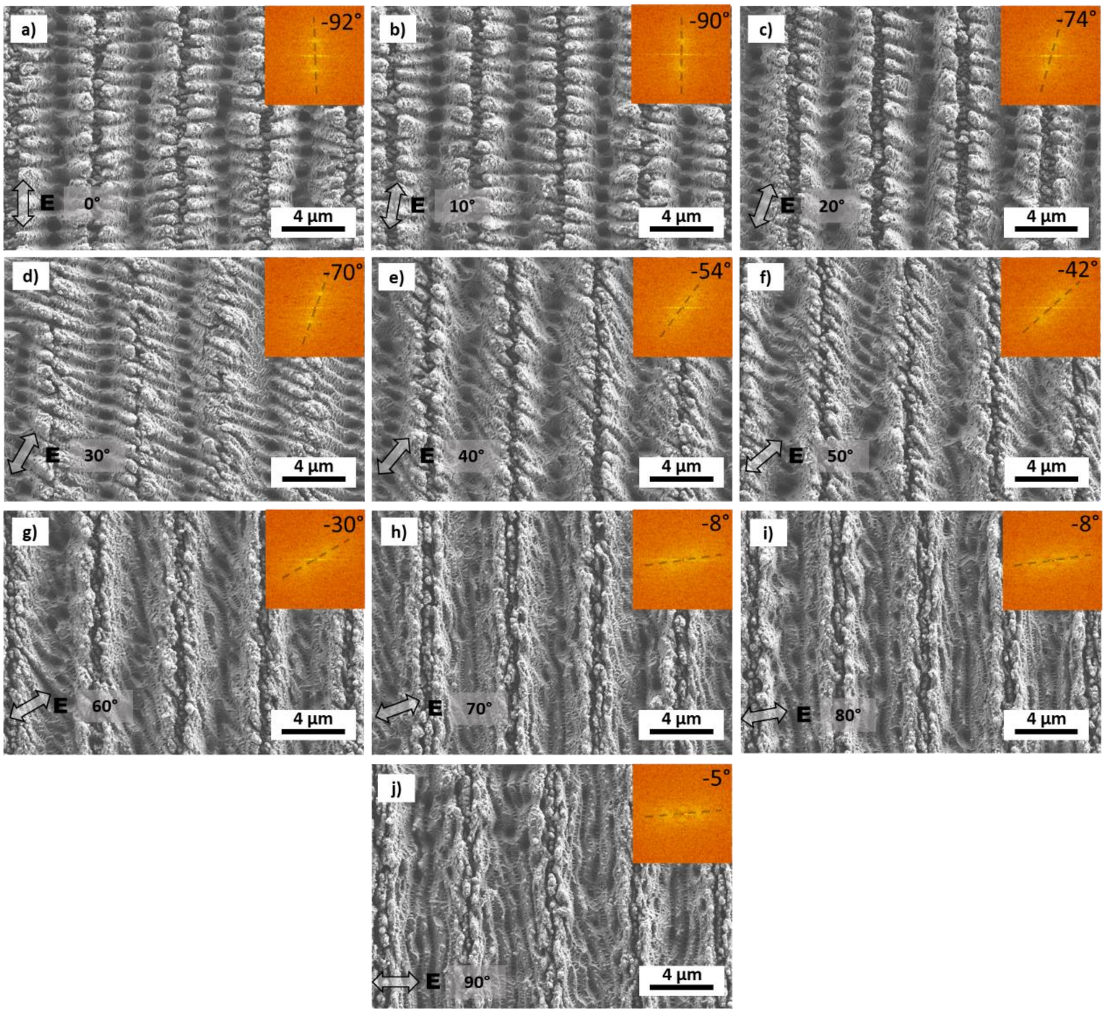

Figure S2: SEM images of DLIP Stainless Steel surfaces treated with 70 ps pulses and 160.0 J/cm² fluence at different polarisation orientation angles; (a) 0°; (b) 10°; (c) 20°; (d) 30°; (e) 40°; (f) 50°; (g) 60°; (h) 70°; (i) 80°; j (90°). The insets show the calculated FFT corresponding to the SEM image, as well as the calculated oientation angle for the LSFL.

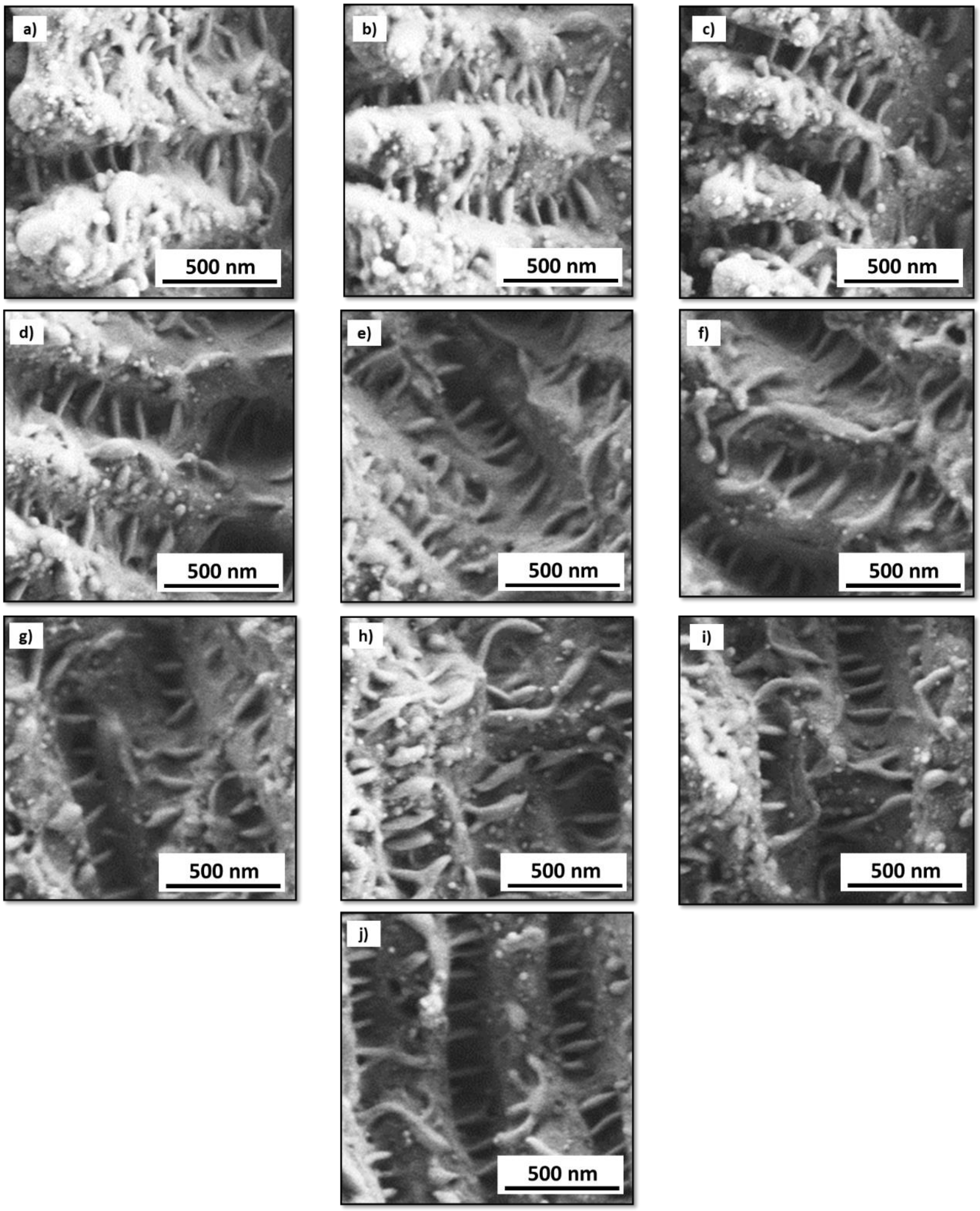

Figure S3: High resolution SEM images showing the HSFL formed perpendicular to the LSFL in stainless steel surfaces treated with 70 ps pulses and 160.0 J/cm² fluence. The corresponding polarization angles are: (a) 0°; (b) 10°; (c) 20°; (d) 30°; (e) 40°; (f) 50°; (g) 60°; (h) 70°; (i) 80°; j (90°).

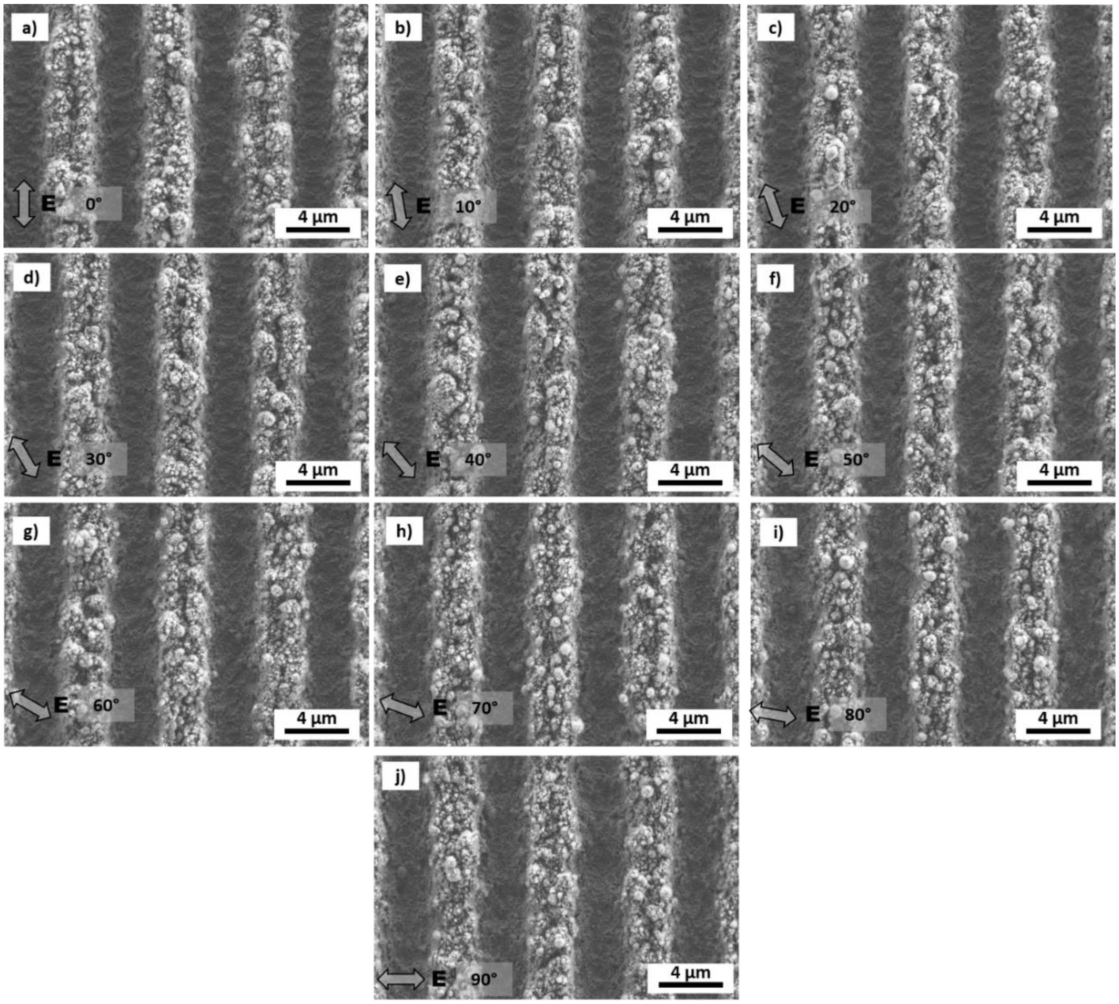

Figure S3: DLIP Al 1024 surfaces treated with 12 ps pulses and 22.4 J/cm² fluence at different polarisation orientation angles; (a) 0°; (b) 10°; (c) 20°; (d) 30°; (e) 40°; (f) 50°; (g) 60°; (h) 70°; (i) 80°; j (90°).

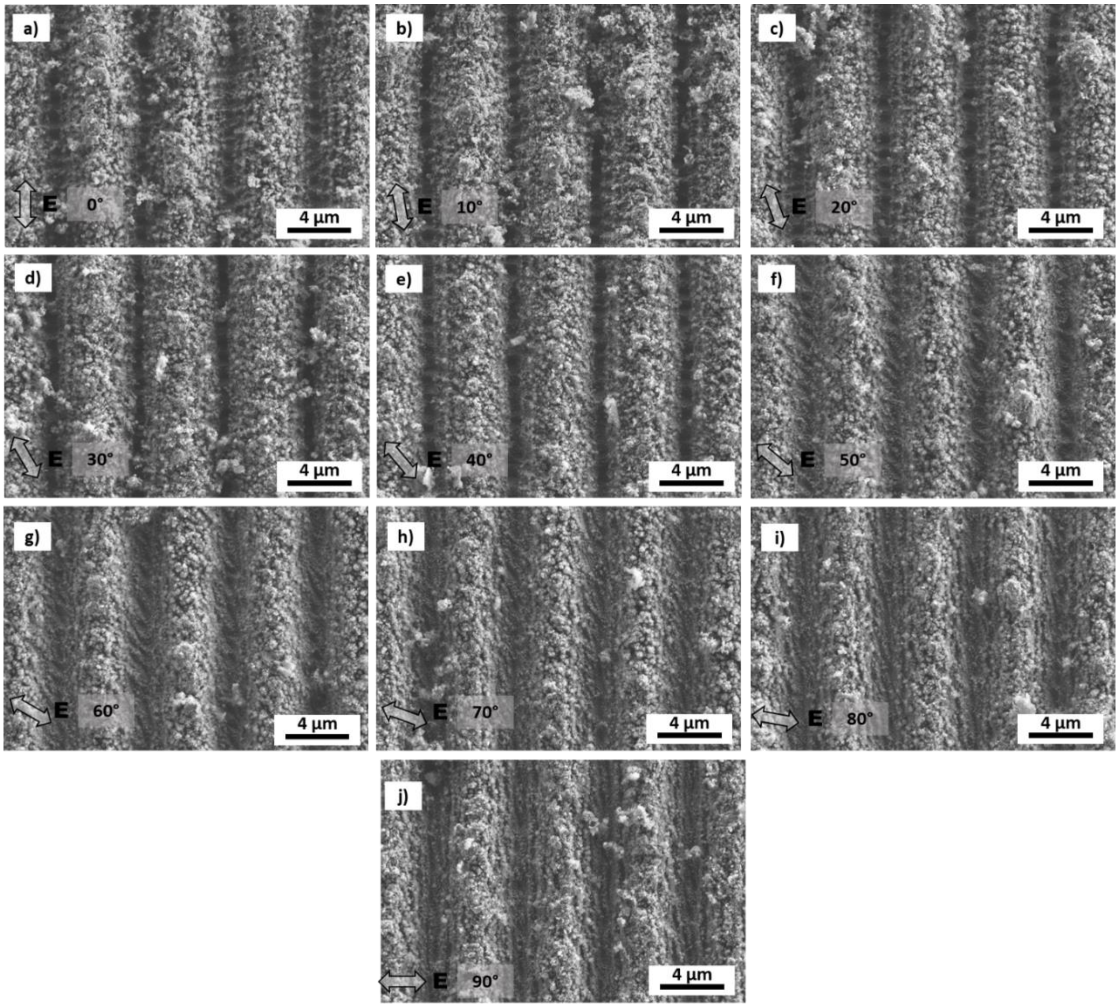

Figure S4: DLIP Al 1024 surfaces treated with 70 ps pulses and 160.0 J/cm² fluence at different polarisation orientation angles; (a) 0°; (b) 10°; (c) 20°; (d) 30°; (e) 40°; (f) 50°; (g) 60°; (h) 70°; (i) 80°; j (90°).
